# Signaling Pathways in Cardiac Myocyte Apoptosis

**DOI:** 10.1155/2016/9583268

**Published:** 2016-12-22

**Authors:** Peng Xia, Yuening Liu, Zhaokang Cheng

**Affiliations:** Department of Pharmaceutical Sciences, Washington State University College of Pharmacy, PBS 323, 205 E. Spokane Falls Blvd., P.O. Box 1495, Spokane, WA 99210-1495, USA

## Abstract

Cardiovascular diseases, the number 1 cause of death worldwide, are frequently associated with apoptotic death of cardiac myocytes. Since cardiomyocyte apoptosis is a highly regulated process, pharmacological intervention of apoptosis pathways may represent a promising therapeutic strategy for a number of cardiovascular diseases and disorders including myocardial infarction, ischemia/reperfusion injury, chemotherapy cardiotoxicity, and end-stage heart failure. Despite rapid growth of our knowledge in apoptosis signaling pathways, a clinically applicable treatment targeting this cellular process is currently unavailable. To help identify potential innovative directions for future research, it is necessary to have a full understanding of the apoptotic pathways currently known to be functional in cardiac myocytes. Here, we summarize recent progress in the regulation of cardiomyocyte apoptosis by multiple signaling molecules and pathways, with a focus on the involvement of these pathways in the pathogenesis of heart disease. In addition, we provide an update regarding bench to bedside translation of this knowledge and discuss unanswered questions that need further investigation.

## 1. Introduction

Cell survival and death are fundamental for organ development, tissue homeostasis, and disease pathogenesis. Based on morphological manifestations, cell death was initially classified into three categories by Schweichel and Merker: type I (apoptosis) that is associated with cell fragmentation and heterophagy, type II (autophagic cell death) that is characterized by massive cytoplasmic vacuolization, and type III (necrosis) that is characterized by plasma membrane rupture and organelle swelling [1]. Since Kerr and colleagues introduced the concept of “apoptosis” to the scientific community in 1972 [2], the knowledge regarding this specific type of cell death has expanded tremendously. The original definition of apoptosis was based on morphological characteristics including chromatin condensation, nuclear fragmentation, cell shrinkage, and shedding of vacuoles (apoptotic bodies) that are eventually cleared via phagocytosis in vivo. However, with the substantial progress in the molecular mechanisms underlying cell death in the past decade, the Nomenclature Committee on Cell Death (NCCD) recently recommended defining various types of cell death based on their distinct biochemical characteristics [3]. In this regard, apoptosis is defined as a caspase-dependent, genetically controlled form of cell death [3]. This definition indicates that apoptosis is a biological process that can be modulated by genetic or pharmacologic interventions. In addition to apoptosis, multiple forms of regulated cell death exist, such as autophagic cell death that is associated with lipidation of microtubule-associated protein light chain 3 (LC3) and degradation of sequestosome 1 (SQSTM1, also known as p62) [4] and necroptosis that is dependent on receptor-interacting protein kinases 1 and 3 (RIPK1/RIPK3) [5]. Interested readers are referred to the above review articles that discuss each cell death modality more comprehensively. Here we focus on the apoptotic form of cell death.

**Figure 1:**
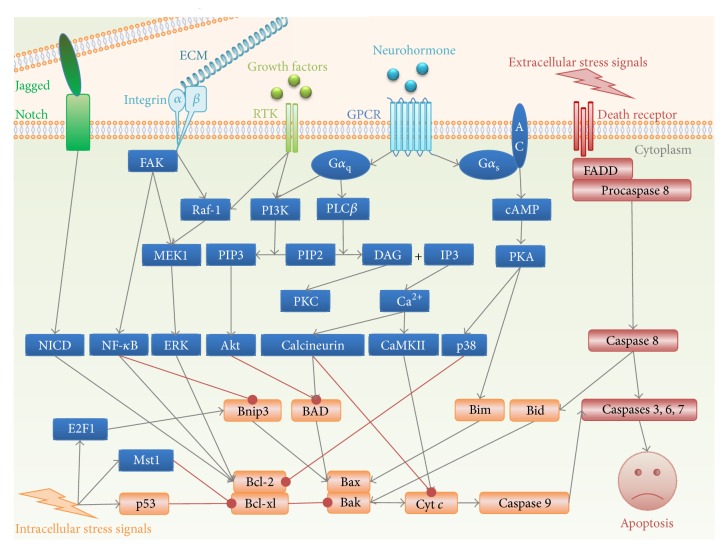
Schematic representation of the signaling pathways that regulate apoptosis in mammalian cardiac myocytes. AC, adenylyl cyclase; CaMKII, Ca^2+^/CaM-dependent kinase II; cAMP, cyclic adenosine monophosphate; Cyt* c*, cytochrome c; DAG, diacylglycerol; E2F1, E2F transcription factor 1; ECM, extracellular matrix; ERK, extracellular signal-regulated kinase; FADD, Fas-associated death domain; FAK, focal adhesion kinase; GPCR, G protein-coupled receptor; IP3, inositol 1,4,5-trisphosphate; MEK1, mitogen-activated protein kinase 1; Mst1, mammalian Ste20-like kinase 1; NICD, Notch intracellular domain; PI3K, phosphoinositide 3-kinase; PIP2, phosphatidylinositol bisphosphate; PIP3, phosphatidylinositol trisphosphate; PLC*β*, phospholipase C *β*; PKA, cyclic AMP-dependent protein kinase; PKC, protein kinase C; RTK, receptor tyrosine kinase. Gray arrows indicate activation; red blocked lines indicate inhibition.

Apoptosis is initiated and executed through two major signaling pathways: the intrinsic and extrinsic pathways. Intrinsic apoptosis pathway (also named as mitochondrial apoptosis pathway) is triggered by intracellular stress such as oxidative stress, calcium overload, and DNA damage, leading to Bax/Bak-dependent mitochondrial outer membrane permeabilization (MOMP) and release of cytochrome* c* from mitochondria into cytosol (Figure 1). Cytosolic cytochrome* c* and apoptotic protease-activating factor 1 (Apaf-1) then form apoptosome and result in activation of caspase 9. By contrast, extrinsic apoptosis is initiated by extracellular stress signals including tumor necrosis factor-*α* (TNF-*α*), Fas ligand (FasL), and TNF-related apoptosis inducing ligand (TRAIL) through binding to their individual death receptors TNF-*α* receptor 1 (TNFR1), Fas, and TRAIL receptor 1/2 (TRAILR1/2), respectively. Death receptors then recruit Fas-associated death domain (FADD) and procaspase 8 into the death-inducing signaling complex (DISC), leading to caspase 8 activation (Figure 1). The activated initiator caspase 9 or 8 further induces activation of the effector caspases 3, 6, and 7, resulting in cleavage of essential cellular substrates and eventually apoptotic death of the cell.

Apoptosis is essential during heart development and has long been linked to a number of cardiovascular diseases such as ischemic heart disease, reperfusion injury, chemotherapy-induced cardiomyopathy, and heart failure [6]. Significant apoptosis was detected in embryonic heart at the time of outflow tract shortening [7], and inhibition of apoptosis using the pan-caspase inhibitor zVAD-fmk or adenoviral mediated expression of X-linked inhibitor of apoptosis protein resulted in excessive outflow tract above the base of the ventricles [8], indicating that apoptosis is required for morphogenesis of the outflow tract myocardial tissue. Although apoptosis appears to be dispensable for physiological homeostasis in normal adult heart, it can lead to cardiomyocyte loss that is associated with life-threatening cardiac dysfunction in multiple pathological settings [9, 10]. Therefore, modulation of apoptosis is a promising therapeutic strategy for cardiovascular diseases.

Cardiac myocytes, which represent ~85% of total heart mass, are the major contracting cells in the heart. In the past decade, major progress has been made toward understanding the apoptosis mechanisms in cardiomyocytes. We feel that a comprehensive review of these new discoveries is urgently needed in the field. In this review, we will summarize the signaling pathways that regulate cardiomyocyte apoptosis, highlight important new findings, and discuss potential future directions of research.

## 2. PI3K/Akt Pathway

PI3K/Akt signaling pathway is activated following stimulation with various growth factors, cytokines, and hormones. Upon ligand binding, growth factor receptors, which are a group of receptor tyrosine kinases (RTKs), undergo dimerization and association with the regulatory subunit (p85) of PI3K, leading to activation of the catalytic subunit (p110, Figure 1). PI3K then converts phosphatidylinositol bisphosphate (PIP2) to phosphatidylinositol trisphosphate (PIP3), which recruits Akt to the plasma membrane, where it is activated through dual phosphorylation by PDK1 at Thr308 and by mTORC2 at Ser473. The role of Akt in myocardial biology has been extensively described by us in a previous review article [11]. Here we briefly summarize the recent findings about this pathway in regulation of apoptosis in cardiomyocytes.

The PI3K/Akt pathway was initially shown to be strongly activated by insulin and insulin-like growth factor 1 (IGF-1) and mediates its antiapoptotic effects in cardiomyocytes since blockade of this pathway by a specific PI3K inhibitor wortmannin, dominant-negative PI3K, or dominant-negative Akt dramatically inhibited the cytoprotective effect of insulin and IGF-1 [12, 13]. PI3K/Akt-mediated protection against apoptosis is associated with phosphorylation and inactivation of the BH3-only proapoptotic protein BAD (Figure 1) [13]. In vivo gene transfer of myristoylated Akt (myr-Akt), a membrane-localized constitutively active Akt1 mutant, also increased sarcolemmal Glut-4 expression and enhanced myocyte glucose uptake and glycolysis which may help maintain energy production in the oxygen-deprived ischemic heart [14]. Intriguingly, while acute moderate Akt1 activation is cardioprotective [12, 14, 15], chronic extensive activation of Akt1 in a cardiac-specific myr-Akt transgenic mice is deleterious during ischemia/reperfusion (I/R) due to feedback inhibition of PI3K activity through downregulation of insulin receptor substrate-1 (IRS-1) and IRS-2 [16]. These findings suggest that the timing and dose of activation are crucial for the effect of Akt on heart protection in vivo.

Subcellular localization of Akt also modulates its biological function likely through altering access to distinct substrates. Physiological importance of nuclear Akt was originally proposed based on the observation that the cardioprotective hormone estrogen is associated with increased nuclear phospho-Akt (Ser473) in human hearts and cultured cardiomyocytes [17]. Overexpression of nuclear-targeted Akt protected against myocardial I/R injury and cardiomyocyte apoptosis without phosphorylating typical cytoplasmic Akt substrates [18]. The myocardial target of nuclear Akt identified so far includes forkhead transcription factors [17], zyxin [19], and Pim1 [20]. Mitochondrial translocation of Akt was also documented in cardiomyocytes following treatment with leukemia inhibitory factor (LIF) [21]. At mitochondria, Akt phosphorylates hexokinase-II at Thr473 to protect outer membrane integrity through a mechanism that is still largely unclear [22].

Both Akt family members Akt1 and Akt2 are abundantly expressed in the heart. It has been shown that Akt2 also plays a cardioprotective role as genetic deficiency of Akt2 exaggerated cardiomyocyte apoptosis in response to ischemic injury [23]. The importance of the PI3K/Akt pathway in cardiomyocyte survival is further supported by its involvement in cardioprotection conferred by hypoxic preconditioning [24, 25], 17beta-estradiol [26], leukemia inhibitory factor (LIF) [27], neuregulin-1 [28], adrenomedullin [29], kallikrein [30], isoflurane [31], clusterin [32], tanshinone IIA [33], urotensin II [34], apoptosis regulator through modulating IAP expression (ARIA) [35], and glucagon-like peptide 1 (GLP1) [36].

### 2.1. PTEN

In contrast to PI3K, the dual protein/lipid phosphatase and tensin homologue (PTEN) dephosphorylates PIP3 to generate PIP2 and thus inhibits Akt activation. It has been shown that adenoviral expression of PTEN in neonatal rat cardiomyocytes led to caspase 3 activation and apoptosis [37]. Conversely, inactivation of PTEN by cardiac-specific deletion of PTEN gene or overexpression of a catalytically inactive PTEN mutant attenuated myocyte apoptosis in response to I/R injury or *β*_1_-AR stimulation [38, 39]. PTEN activity is positively regulated by direct interaction with the regulatory subunit (p85) of PI3K [40]. Indeed, expression of a p85 mutant lacking the PTEN binding site inhibited PTEN activity and cell death following simulated ischemia and reperfusion [41]. PTEN activation is observed in cardiomyocytes expressing a cleaved (and constitutively active) mutant of Rho-associated coiled-coil protein kinase 1 (ROCK1) and may contribute to ROCK1-dependent apoptosis [42].

### 2.2. PHLPP

Akt activity can be inhibited by PH domain leucine-rich repeat protein phosphatase (PHLPP), a protein phosphatase 2C(PP2C) family member that selectively dephosphorylates Akt at Ser473. Knockout of PHLPP-1 potentiated Akt phosphorylation at Ser473 and reduced infarct size in response to I/R challenge [43]. A most recent study revealed that PHLPP-1 expression is increased with aging, and an increase in PHLPP-1 expression exacerbated hypoxia/reoxygenation-induced apoptosis [44]. Similarly, Akt can also be dephosphorylated by PHLPP-2, which is activated by isoproterenol and forskolin through a cyclic AMP- (cAMP-) independent mechanism [45]. However, its role in cardiomyocyte apoptosis has not been studied yet.

### 2.3. GSK-3

Ischemic preconditioning-induced, Akt-dependent phosphorylation and inactivation of its downstream target glycogen synthase kinase-3 (GSK-3) were initially speculated to confer cardioprotection because both preconditioning and pretreatment with GSK-3 inhibitors reduced infarct size to a similar extent [46]. These findings were supported by transgenic studies showing that cardiac-specific expression of either GSK-3*β* or GSK-3*α* potentiated myocyte apoptosis, albeit through distinct mechanisms [47, 48]. Conversely, cardiac-specific deficiency of GSK-3*β* significantly inhibited myocyte apoptosis after myocardial infarction (MI) [49]. Although global deletion of GSK-3*α* exacerbated apoptosis after MI [50], following up studies revealed that cardiomyocyte-specific conditional deletion of GSK-3*α* reduced apoptosis by decreasing the Bax/Bcl-2 ratio [51]. The discrepancy is likely caused by secondary and compensatory effects associated with germline somatic gene deletion. Surprisingly, a most recent study revealed that conditional deletion of cardiac GSK-3*α*/GSK-3*β* in adult mice resulted in ventricular dysfunction and dilation within 2 weeks through a mechanism involving DNA damage and mitotic catastrophe [52]. Collectively, these studies indicate that transient and partial inhibition of GSK-3 is cardioprotective, but complete loss of GSK-3 leads to dilated cardiomyopathy.

### 2.4. Pim1

An important downstream target of myocardial Akt signaling has been demonstrated to be Pim1, a serine-threonine kinase that is robustly upregulated following treatment with the cardioprotective growth factor IGF-1 through an Akt-dependent mechanism [20]. Inhibition of Pim1 activity by genetic ablation of Pim1 or expression of a dominant-negative, kinase-dead Pim1 mutant (K67M) exacerbated cardiomyocyte apoptosis, whereas transgenic expression of Pim1 markedly reduced infarct size in mice [20, 53]. Further in-depth studies revealed that cardiac-specific Pim1 expression increased levels of the prosurvival proteins Bcl-2 and Bcl-xl, which antagonized mitochondrial damage induced by oxidative stress and the proapoptotic truncated Bid protein [54]. Pim1-dependent inhibition of apoptosis has been implicated in cardioprotection induced by postconditioning and vitamin B1 stimulation [55, 56], supporting a critical role of Pim1 in regulation of cardiomyocyte survival following pathological challenge.

### 2.5. FoxO1

Akt phosphorylates the FoxO family transcription factors, resulting in their nuclear export and degradation in the cytosol through the ubiquitin-proteasome pathway [57]. FoxO has long been viewed as proapoptotic through transcriptional induction of proteins involved in intrinsic and extrinsic apoptosis pathways including Bim, BAD, Bnip3, FasL, and TRAIL [57]. Interestingly, overexpression of FoxO1 in cardiomyocytes did not seem to result in apoptosis under basal conditions but significantly induced expression of autophagy-related genes LC3 and Atg12 and enhanced autophagy [58, 59]. Following MI, however, cardiac-specific deficiency of FoxO1 decreased heart function and increased myocyte apoptosis, an effect that is associated with reduced expression of autophagy genes [60]. In addition, FoxO1 may also protect against apoptosis by forming a transcriptional complex with Yes-associated protein (YAP) and inducing expression of antioxidant genes catalase and manganese superoxide dismutase (MnSOD) [60, 61]. Moreover, another FoxO family member, FoxO3a, has also been shown to inhibit cardiomyocyte apoptosis and confers cardioprotection by inducing expression of apoptosis repressor with caspase recruitment domain (ARC), which attenuated oxidative stress-triggered sarcoplasmic reticulum Ca^2+^ release [62]. A most recent study showed that knockdown of FoxO1 that is exported from the nuclei following Apelin-13 stimulation exaggerated apoptosis, suggesting that cytosolic FoxO1 may directly inhibit apoptosis through a transcription activity-independent mechanism [63].

## 3. MAPK Pathway

Mitogen-activated protein kinases (MAPKs) are evolutionarily conserved serine/threonine kinases that regulate cell behavior including survival, growth, and differentiation by altering protein function and gene expression in response to specific extracellular cues. Extracellular signals act through cell surface receptors such as RTKs and G protein-coupled receptors (GPCRs), leading to successive phosphorylation and activation of a three-layered hierarchical model including MAPK kinase kinase (MAPKKK), MAPK kinase (MAPKK), and MAPK. Three main families of MAPKs have been extensively investigated in regulation of cardiomyocyte apoptosis: extracellular signal-regulated kinase 1/2 (ERK1/2 or p44/42), c-Jun N-terminal kinases (JNK), and the p38 isoforms.

### 3.1. ERK1/2

ERK1/2 is activated following growth factor stimulation or integrin clustering, and activation of ERK1/2 primarily leads to cell growth and survival (Figure 1). ERK1/2 may also be activated by hydroxyl radicals through Ras/Raf-1 MAPKKK, but activation of ERK in this context is still protective as treatment with a selective ERK inhibitor PD98059 exacerbated hydrogen peroxide- (H_2_O_2_-) induced cardiomyocyte apoptosis [64]. Indeed, while all three MAPKs are activated following daunomycin treatment and hypoxia/reoxygenation, only inhibition of ERK1/2 further potentiated cardiomyocyte apoptosis, suggesting a prosurvival role of the ERK1/2 kinases [65, 66]. Interestingly, doxorubicin-induced persistent ERK1/2 activation and nuclear translocation contributed to apoptosis in H9c2 cells and neonatal rat cardiomyocytes [67]. Inactivation of ERK1/2 by transgenic expression of a dominant-negative Raf-1, the MAPKKK upstream of ERK1/2, potentiated development of cardiomyocyte apoptosis following aortic constriction [68]. Mice deficient in cardiac Raf-1 exhibited spontaneous myocyte apoptosis as early as 3 weeks of age and heart dysfunction later in life [69]. Transgenic mice expressing a constitutively active MEK1, the major MAPKK directly phosphorylating and activating ERK1/2, inhibited I/R-induced cardiomyocyte apoptosis [70]. A more direct evidence of ERK-dependent cardioprotection was from the observation that myocytes were sensitized to apoptosis following pathological insult in both global and cardiac-specific ERK2 knockout mice [70, 71]. Activation of ERK1/2 has been shown to mediate the antiapoptotic function of various molecules including *α*1-adrenergic receptor [72], urotensin II [34], and sialyltransferase7A [73]. However, much less is known about the mechanism(s) underlying ERK1/2-mediated protection against myocyte apoptosis.

### 3.2. Stress Activated Protein Kinases (SAPKs)

In contrast to ERK1/2, JNK and p38 are stress activated protein kinases (SAPKs) that respond predominantly to environmental stress signals such as hypoxia, heat, inflammatory cytokines, and DNA-damaging agents. Inhibition of JNK protected against I/R-induced cardiomyocyte apoptosis in vitro and in vivo [74, 75]. Mitochondrial but not cytosolic JNK was phosphorylated upon H_2_O_2_ stimulation, leading to mitochondrial outer membrane permeabilization and cytochrome* c* release [76]. JNK-dependent activation of the mitochondrial apoptosis pathway is associated with decreased BAD phosphorylation at Ser112 [77, 78]. Inactivation of JNK contributed to the antiapoptotic effect of macrophage migration inhibitory factor (MIF) and a Curcumin analog [78, 79]. While mice deficient in JNK1/2 were shown to be resistant to I/R-induced apoptosis, cardiac-specific transgenic mice expressing MKK7, the MAPKK for JNK kinases, were also protected from I/R injury [77]. Although somewhat surprising, the latter finding was consistent with previous reports revealing a protective role of JNK activation during nitric oxide- or hypoxia/reoxygenation-induced cardiomyocyte apoptosis [80–82], possibly by phosphorylating Akt at Thr450, thus priming Akt for full activation [83], or by competing with procaspase 9 to bind Apaf-1 and abrogating apoptosome formation [84]. These contradictory results indicate that the role of JNK signaling in apoptosis is likely context dependent and much more complicated than initially thought.

Earlier studies revealed that treatment with the p38 MAPK inhibitor SB203580 attenuated myocyte apoptosis induced by anthracycline and hypoxia/reoxygenation both in vitro and in vivo, indicating a detrimental role of p38 [65, 66, 85, 86]. These findings were later corroborated by using genetic approaches to inactivate p38 in mouse heart. For example, heterozygous disruption of p38*α* drastically reduced infarct size after I/R challenge [87]. Cardiomyocyte apoptosis and cardiac dysfunction following I/R were also attenuated by p38 inactivation in transgenic mice expressing a dominant-negative p38*α* mutant, a dominant-negative MKK6 (the MAPKK upstream of p38) mutant, or MAPK phosphatase-1 (MKP-1, a phosphatase that dephosphorylates and inactivates p38 and JNK) [88, 89]. Cardioprotection by p38 inhibition was associated with an increase in the expression of the prosurvival Bcl-2 family members Bcl-2/Bcl-xl [88, 89]. Although p38 is activated by short repeated cycles of simulated ischemia/reoxygenation termed ischemic preconditioning, preconditioned heart actually exhibited lower p38 activity and was protected against injury during sustained ischemia [90, 91], likely through p38-dependent feedback upregulation of MKP-1 [92]. Inhibition of p38 activity has been proposed as a central mechanism underlying cardioprotection mediated by postconditioning [93], estrogen [94], and quercetin [95].

## 4. Integrin/FAK

Integrins are transmembrane heterodimers of *α* and *β* subunits that physically link extracellular matrix (ECM) to intracellular actin cytoskeleton. In addition to maintaining cell adhesion capability and tissue integrity, integrins also transmit outside mechanical signals into the cell (outside-in signaling). Israeli-Rosenberg et al. recently provided a detailed review of integrins' multiple functions in cardiac myocytes [96] and hence we will only focus on their regulation of apoptosis signals.

The dominant *β* subunit in heart is *β*1 integrin, which forms a heterodimer with *α*1, *α*5, or *α*7 to predominantly bind ECM components collagen, fibronectin, or laminin, respectively. Global homozygous loss of *β*1 integrin, as well as embryonic cardiac-specific knockout of *β*1 integrin (*β*1 integrin^flox/flox^/Nkx2.5^Cre/+^), was embryonically lethal [97–99]. However, the *β*1 integrin heterozygous knockout mice developed into adulthood and were more vulnerable to cardiac dysfunction and myocyte apoptosis upon induction of MI or stimulation with the *β* adrenergic receptor agonist isoproterenol [100, 101], suggesting that *β*1 integrin protects myocyte against apoptosis in these settings. It is noteworthy that pressure overload did not appear to unmask the antiapoptotic role of *β*1 integrin in acute (*β*1 integrin^flox/flox^/*α*-MHC Mer-Cre-Mer) or chronic myocyte-specific knockout models (*β*1 integrin^flox/flox^/Mlc2v^Cre/+^) [102, 103]. The fact that acute or chronic ablation of *β*1 integrin in cardiomyocytes depressed ventricular contractility and blunted hypertrophic response without inducing apoptosis indicates that *β*1 integrin may protect the heart via additional mechanisms that are independent of its role in apoptosis inhibition. Nonetheless, it would still be interesting to evaluate whether cardiac-specific depletion of *β*1 integrin modulates responses to I/R or *β* adrenergic signaling-induced apoptosis, especially with the exciting observation that overexpression of integrin *α*7*β*1D attenuated I/R injury and promoted cardiomyocyte survival [104].

Since integrins do not have an intracellular kinase domain, integrin-dependent signal transduction requires cytosolic kinases including focal adhesion kinase (FAK). Based on earlier studies demonstrating that myocyte-restricted deletion of FAK exacerbated myocardial I/R injury [105], we demonstrated that cardiac-specific activation of FAK protected cardiomyocytes from I/R-induced apoptosis by enhancing NF-*κ*B-dependent transcription of the prosurvival Bcl-2 family members Bcl-2 and Bcl-xl and X-linked inhibitor of apoptosis (XIAP, Figure 1) [106]. In addition, FAK also confers protection against anthracycline doxorubicin-induced cardiomyopathy and myocyte apoptosis through increasing mRNA stability of the cyclin-dependent kinase (CDK) inhibitor (CDKI) p21 [107]. Our findings that p21 levels were inversely correlated with mitochondrial vulnerability and expression of the BH3-only protein Bim following doxorubicin treatment suggested that mitochondrial apoptosis pathway was inhibited by FAK/p21. Exactly how p21 regulates Bim expression in cardiomyocytes warrants further investigation.

## 5. TNF-***α***/NF-***κ***B

Tumor necrosis factor-*α* (TNF-*α*), a 17 kDa proinflammatory cytokine generated after cleavage of the 26 kDa transmembrane protein pro-TNF-*α* by TNF-*α* converting enzyme (TACE), is secreted from resident mast cells and macrophages within minutes of myocardial I/R injury through a mechanism that is dependent on oxidative stress [108]. While cardiac-restricted expression of a nonsecretable transmembrane TNF-*α* mutant led to concentric hypertrophy, expression of the secreted wild-type TNF-*α* using the same strategy resulted in ventricular dilation [109], which is likely caused by primary activation of the extrinsic apoptosis pathway and subsequent t-Bid-dependent secondary activation of the intrinsic mitochondrial apoptosis pathway [110], caspase-dependent desmin cleavage and mislocalization [111], and NF-*κ*B-dependent transcriptional suppression of sarcoplasmic reticulum Ca^2+^-ATPase 2a protein (SERCA2a) [112]. Consistently, global deficiency of TNF-*α* reduced inflammatory cell infiltration and cardiac rupture after permanent ischemia or I/R injury [113, 114]. The fact that deletion of TNF-*α* receptor 1 (TNFR1), but not TNFR2, protected the heart against I/R injury suggested that the detrimental effect of TNF-*α* was likely mediated by TNFR1 [115, 116]. Indeed, studies from an independent group revealed that knockout of TNFR1 attenuated apoptosis and suppressed activation of NF-*κ*B, p38, and JNK2 in a permanent ligation model, while deletion of TNFR2 augmented apoptosis and enhanced activation of NF-*κ*B and p38, suggesting that activation of TNFR1 and TNFR2 mediates detrimental and beneficial signals, respectively [117].

Given the divergent roles of TNFR1 and TNFR2, it is not surprising that TNF-*α* may exert cytoprotection in some context [118, 119]. It has been shown that TNF-*α* protected against myocardial I/R injury through TNF receptor-associated factor 2- (TRAF2-) dependent activation of NF-*κ*B [120], leading to enhanced transcription of prosurvival Bcl-2 and c-IAP1 [121], transcriptional silencing of the death gene Bnip3 [122], or expression of keratin 8 and keratin 18 [123]. Moreover, inactivation of NF-*κ*B by deletion of NF-*κ*B essential modulator (NEMO)/inhibitor of *κ*B kinase (IKK)*γ* led to spontaneous ventricular dilation that is associated with exaggerated apoptosis and decreased expression of Bcl-xl [124]. It is noteworthy that the protective role of NF-*κ*B may only be limited in cases where the activation of NF-*κ*B is acute and transient, as persistent NF-*κ*B activation by cardiomyocyte-specific expression of a constitutively active IKK2 led to inflammatory dilated cardiomyopathy, a phenotype that is associated with enhanced activation of the IFN-stimulated gene 15 (ISG15) pathway, and could be reversed by in vivo expression of the endogenous NF-*κ*B inhibitor I-*κ*B*α* [125]. Chronic NF-*κ*B p65 activation in the remote myocardium after permanent ligation has been linked to exacerbated apoptosis and myocardial dilation and dysfunction [126], and cardiomyocyte-restricted NF-*κ*B inhibition in transgenic mice expressing the nonphosphorylatable I-*κ*B*α* (S32A, S36A) or I-*κ*B*α* (S32A, S36A, and Y42F) or the p65-deficient mice conferred cardioprotection [126–128].

In addition to the Rel subfamily members (p65/RelA, RelB, and c-Rel) that contain a transcriptional activating domain (TAD), NF-*κ*B family transcription factors also consist of a “NF-*κ*B” subfamily (p50 and p52) without a TAD. The role of p50 in regulation of cardiomyocyte apoptosis has been controversial based on data generated from global p50 knockout mice, with deleterious [129, 130] and protective [131] effects being reported. The discrepancy may be explained by the fact that p50 can activate transcription when dimerization with p65 occurs but may also serve as an endogenous NF-*κ*B inhibitor when it forms a p50/p50 homodimer [132].

## 6. GPCR

G protein-coupled receptors (GPCRs), the largest gene family in the genome, are conserved seven transmembrane receptors that have been extensively targeted for drug therapy. Ligand binding to GPCR induces dissociation of the G protein subunits G*βγ* with G*α*_s_, G*α*_i_, G*α*_q_, or G*α*_11/12_, leading to divergent physiological responses. Dissociated G*βγ* targets GPCR kinase 2 (GRK2) to the membrane and promotes GPCR desensitization. GRK2-mediated desensitization of adiponectin receptor 1 exacerbated ventricular remodeling after MI [133]. GRK2 was also localized to mitochondria following ischemic injury to induce opening of the mitochondrial permeability transition pore (mPTP) and cell death [134]. Consistently, depletion of GRK2 in cardiomyocytes suppressed cardiomyocyte apoptosis and I/R injury [135]. The best investigated cardiac GPCRs include G*α*_s_- and G*α*_i_-coupled *β*-adrenergic receptors (*β*-ARs); G*α*_q_-coupled *α*1-AR, endothelin receptors, and angiotensin receptors; and G*α*_i_-coupled *α*2-AR and adenosine receptors [136].

The predominant forms of cardiac ARs are *β*1-AR (~70%), *β*2-AR (~20%), and *α*1-AR (~10%) [137]. Stimulation of *β*-ARs induces G*α*_s_-mediated activation of adenylyl cyclase, leading to production of cAMP and activation of cAMP-dependent protein kinase (PKA, see below for details). Adult rat cardiomyocytes treated with *β*-AR agonists isoproterenol or norepinephrine underwent apoptosis that can be inhibited by *β*1-AR antagonist but was potentiated by *β*2-AR antagonist, suggesting a detrimental role of the G*α*_s_-coupled *β*1-AR and a beneficial role of the G*α*_i_-coupled *β*2-AR [138, 139]. Stimulation of *β*1-AR induced cardiomyocyte apoptosis by activation of PKA [140], calcineurin [141], Ca^2+^/calmodulin kinase II (CaMKII) [142], and reactive oxygen species-dependent synthesis of TNF-*α* [143], whereas stimulation of *β*2-AR suppressed cardiomyocyte apoptosis by activation of the PI3K/Akt pathway [144–146]. Consistently, transgenic mice selectively expressing *β*1-AR-associated G protein G*α*_s_ in the heart displayed aging-related cardiomyopathy due to increased myocyte apoptosis [147], while cardiac-specific inhibition of the *β*2-AR-associated G*α*_i_ by transgenic expression of G*α*_i_ inhibitor peptide provoked apoptosis and exacerbated myocardial I/R injury [148]. However, it is noteworthy that stimulation of *β*2-AR by isoproterenol has also been associated with enhanced apoptosis via PKA-mediated activation of p38 MAPK in some contexts [149, 150]. A possible explanation is that isoproterenol may promote cell survival or death in a dose-dependent fashion via activation of distinct pathways: treatment with low-dose isoproterenol enhances cell survival by increasing ERK1/2-mediated expression of Bcl-2, while stimulation with high concentration of isoproterenol leads to cell death through PKA-dependent downregulation of Bcl-2 [140]. Interestingly, chronic *β*1-AR stimulation by transgenic expression of *β*1-AR or by isoproterenol injection also resulted in necrotic myocyte death that is dependent on mammalian Ste20-like kinase 1 (Mst1) [151].

Stimulation of *α*1-AR with the *α*1-AR agonist phenylephrine protected against cardiomyocyte apoptosis induced by a number of pathological stimuli such as isoproterenol [152], hypoxia [153], and doxorubicin [154]. By contrast, cardiomyocytes deficient in *α*1-AR were more susceptible to stress-induced apoptosis [155]. Cardiac myocytes express two *α*1-AR protein subtypes, *α*1A-AR and *α*1B-AR, in a ratio of 2–4 : 1 [137]. Vulnerability caused by *α*1-AR deletion was rescued by reintroduction of *α*1A-AR but not *α*1B-AR through activation of ERK [72]. Intriguingly, sustained supraphysiological activation of *α*1A-AR (66-fold increase) provoked apoptosis and induced premature death of animals [156]. These results are in agreement with previous observation that while expression of the wild-type *α* subunit of Gq leads to Akt activation and cytoprotection [157], expression of a constitutively active G*α*_q_ subunit (GqQ209L) paradoxically reduced Akt activity and induced apoptosis due to exhaustion of cellular PIP2 pool caused by hyperactivation of phospholipase C *β* (PLC*β*, Figure 1) [158]. Under stress conditions such as pregnancy which may further enhance G*α*_q_ activity, cardiac-specific expression of wild-type G*α*_q_ induced apoptosis through a ROCK1-dependent mechanism [159]. Taken together, these studies indicate that moderate activation of the *α*1A-AR/G*α*_q_/ERK pathway is cardioprotective.

The role of endothelin-1 receptor in cardiomyocyte apoptosis has been controversial. Early in vitro studies suggested that stimulation with endothelin-1 protected against isoproterenol-induced cardiomyocyte apoptosis through endothelin type A receptor (ET_A_R) but not type B receptor [160]. However, treatment with the ET_A_R antagonist attenuated myocyte apoptosis in cultured cells [161] and in animal models of MI [162], I/R [163], or doxorubicin-induced cardiomyopathy [164], indicating that endogenous endothelin-1 and ET_A_R are proapoptotic under these conditions. Mice deficient in cardiac endothelin-1 exhibited decreased hypertrophy and increased apoptosis following transverse aortic constriction [165], whereas cardiac-specific deletion of ET_A_R attenuated cold stress-induced hypertrophy and apoptosis [166]. The discrepancy may be caused by differences in dose and timing of drugs and/or pathological insults.

It is known that angiotensin II induces cardiomyocyte apoptosis via angiotensin type 1 receptor (AT1R) but not AT2R [167–169]. Indeed, prolonged activation of cardiac AT1R resulted in increased hypertrophy, fibrosis, and apoptosis [170]. AT1R-mediated apoptosis has been attributed to G*α*_12/13_-induced activation of JNK and p38 MAPK [171], leading to HSF1 acetylation and IGF-IIR expression [172]. Angiotensin II-induced apoptosis can be repressed by activation of the G*α*_i_-coupled A_1_ and A_3_ adenosine receptors [173], which mediates survival signaling through the PI3K/Akt and MEK/ERK1/2 pathways [174, 175].

## 7. Hippo Pathway

The Hippo signaling cascade is an evolutionarily conserved pathway fundamental in organ size control through regulation of cell proliferation, apoptosis, and differentiation. The multifaceted roles of Hippo pathway in cardiovascular development and disease have recently been covered in an excellent review [176]. Mst1, an ortholog of* Drosophila* Hippo, is ubiquitously expressed serine/threonine kinase that has been intensively investigated in enhancing apoptosis in the cardiac settings. Myocardial Mst1 is activated by caspase-mediated cleavage or phosphorylation in response to pathological stimuli that induce genotoxic and oxidative stress [177]. The Sadoshima group has demonstrated that cardiac-specific transgenic expression of a dominant-negative Mst1 mutant (K59R) reduced TUNEL labeling and DNA laddering following I/R or permanent ligation, suggesting that Mst1 is necessary for I/R or MI-induced apoptosis [177, 178]. They further showed that cardiomyocyte-restricted expression of Mst1 was sufficient to induce apoptosis and dilated cardiomyopathy [177]. Mst1 phosphorylates Beclin1 at Thr108, leading to sequestration of Bcl-2/Bcl-xl by phospho-Beclin1 and release of Bax to induce apoptosis [179]. Most recent studies revealed that Mst1 may directly phosphorylate Bcl-xl at Ser14, thereby inhibiting Bcl-xl/Bax interaction and inducing Bax activation and apoptotic death of cardiomyocytes [180, 181]. It is noteworthy that although Mst1^−/−^ or Mst2^−/−^ mice developed normally, homozygous disruption of both Mst1 and Mst2 led to increased apoptosis in embryos, a secondary response to placental functional defects in these mice [182]. These findings suggest that Mst1 and Mst2 kinases are necessary for early mouse development.

Activation of Mst1 by oxidative stress requires the scaffold protein Ras-association domain family 1 isoform A (Rassf1A), which binds mitochondrial K-Ras through its Ras-association (RA) domain and Mst1 through a Salvador/Rassf/Hippo (SARAH) domain [180]. Cardiomyocyte-specific Rassf1A transgenic mice exhibited enhanced Mst1 phosphorylation, increased apoptosis, and impaired cardiac function after pressure overload [183]. Systemic ablation of Rassf1A reduced myocyte apoptosis but unexpectedly induced fibrosis and cardiac dysfunction following pressure overload through enhanced secretion of TNF-*α* by cardiac fibroblasts [183]. Indeed, cardiac-specific disruption of Rassf1A inhibited cardiomyocyte apoptosis and preserved LV ejection function after pressure overload [183]. It is most recently shown that I/R-induced Mst1 activation is mediated by neurofibromin 2 (NF2, Merlin), a 4.1, ezrin, radixin, moesin (FERM) domain-containing protein responsible for membrane and actin cytoskeleton association [184]. Cardiac-specific deletion of NF2 protected against I/R injury through activation of the YAP transcription factor, a main effector of the Hippo pathway [184]. In contrast, mTOR pathway negatively regulates Mst1 kinase activity through mTORC2-dependent phosphorylation of Mst1 at Ser438 in the SARAH domain, leading to decreased homodimerization and activity [185]. Cardiac-specific deletion of Rictor, a major mTORC2 component, induced Mst1 activation and provoked apoptosis both at baseline and in response to pressure overload, leading to cardiac dysfunction and dilation [185].

Active Mst1 directly binds and phosphorylates large tumor suppressors 1/2 (Lats1/2), leading to their activation [176]. It has been shown that knockdown of Lats2 inhibited Mst1-induced apoptosis, indicating that Lats2 mediates the proapoptotic effect of Mst1 [186]. Moreover, cardiac-specific transgenic expression of dominant-negative Lats2 suppressed apoptosis induced by transverse aortic constriction [186].

Lats1/2 kinases phosphorylate and inactivate the transcription coactivator YAP through 14-3-3-mediated cytoplasmic retention and casein kinase-dependent degradation [176]. Heterozygous disruption of YAP in cardiomyocytes increased apoptosis after chronic MI in vivo, whereas overexpression of YAP protected cultured cardiomyocytes against H_2_O_2_-induced apoptosis [187], likely through transcriptional upregulation of antioxidant genes catalase and MnSOD [61]. Cardiac-specific transgenic expression of a constitutively active, nuclear-localized YAP mutant (S112A) decreased apoptosis 7 days after experimental MI [188]. However, cardiac-specific expression of the YAP (S127A) mutant that has been associated with increased nuclear localization mitigated myocardial injury due to enhanced cardiomyocyte proliferation without decreasing apoptosis at 5 weeks after MI, indicating that YAP may regulate other cellular behaviors beyond its antiapoptotic role in the heart [189].

## 8. Small GTPases

Activities of the small GTPases Rho, Rac, and Cdc42 are regulated by guanine exchange factors (GEFs) and GTPase-activating proteins (GAPs) downstream of a variety of extracellular and transmembrane molecules such as hormones, growth factors, and GPCRs. RhoA, a Rho subfamily GTPase, has been extensively studied in cardiac pathophysiology. Earlier studies revealed that supraphysiological expression of RhoA leads to heart failure and activation of the mitochondrial apoptosis pathway due to increased Bax expression [190, 191]. In contrast, moderately increased expression of RhoA was cardioprotective against myocyte apoptosis in vitro and I/R damage in vivo via activation of multiple prosurvival kinases including Rho kinase, FAK, PI3K/Akt, and PKD [192, 193]. The beneficial role of RhoA was further supported by the observation that cardiac-specific deletion of endogenous RhoA exacerbated I/R injury [193] and that pharmacological inhibition of RhoA led to caspase 3 activation and apoptosis [194].

A major RhoA effector Rho-associated coiled-coil protein kinase 1 (ROCK1) is cleaved by caspase 3 into a 130 kDa fragment upon apoptosis induction. When expressed in cardiomyocytes, the truncated ROCK1 by itself induced caspase 3 activation and apoptosis in vitro and myocardial fibrosis in vivo [42, 195]. Interestingly, global deficiency of ROCK1 also inhibited myocyte apoptosis, and transgenic expression of ROCK1 enhanced apoptosis following pressure overload or in a pathological hypertrophy mouse model overexpressing G*α*_q_ [42, 159]. Although not always identical [196], ROCK2 appears to play a similar deleterious role to ROCK1 in apoptosis, as cardiac-specific knockout of ROCK2 reduced oxidative stress, myocyte hypertrophy, and apoptosis following angiotensin II infusion or transverse aortic constriction by inducing expression of four-and-a-half LIM-only protein-2 (FHL2) [197].

Recent studies suggested that the small GTPase Rac plays a detrimental role in cardiomyocyte apoptosis. In a cardiomyocyte-specific Rac1 knockout model generated by breeding Rac1^flox/flox^ mice with *α*-MHC-cre mice, deficiency of Rac1 reduced hyperglycemia-induced mitochondrial ROS production and myocardial dysfunction [198]. Further in vitro and in vivo studies revealed that overexpression of dominant-negative Rac1 or treatment with a Rac1 inhibitor blocked high glucose-induced cardiomyocyte apoptosis and improved heart function in type 2 diabetic db/db mice [198]. Early pioneering studies showed that cardiac-specific transgenic expression of Rac1 resulted in myocardial hypertrophy or dilation depending on temporal windows of expression [199]. In a similar transgenic mouse model, expression of the corn Rac gene led to superoxide generation and caspase activation upon Thyroxin treatment [200]. The observation that Rac-induced apoptosis can be blocked by administration of an antioxidant indicates that oxidative stress plays a causal role in apoptosis in these animals [200].

Another small GTPase, Cdc42, has also been implicated in inhibition of cardiomyocyte apoptosis. Pressure overload activates Cdc42, which acts as a negative feedback mechanism to inhibit hypertrophy through activation of JNK [201]. Cardiac-specific deletion of Cdc42 (Cdc42^flox/flox^/*α*-MHC^Cre/+^) aggravated apoptosis after transverse aortic constriction and may have contributed to hypertrophy decompensation in this model [201].

## 9. PKC

Protein kinase C (PKC) family consists of at least 12 isoforms that mediate hypertrophy and survival signaling in response to growth factors and hormones. Activation of PKC is preceded by phosphorylation of the activation segment by PDK1 and phosphorylation of the hydrophobic motif by mTORC2 [202]. Based on the signals required for their enzymatic activation, PKC isoforms can be divided into conventional PKCs (*α*, *β*, and *γ*) that are calcium and phospholipid dependent, novel PKCs (*δ*, *ε*, *η*, and *θ*) that are calcium independent but phospholipid dependent, and atypical PKCs (*ζ*, *λ*) that are calcium and phospholipid independent. These isoforms appear to have distinct functions as PKC*α* but not PKC*β*; *δ* or *ε* is sufficient and necessary for cardiomyocyte hypertrophy through activation of ERK1/2 [203]. Mice lacking PKC*α* exhibited enhanced cardiac contractility and were resistant to heart failure after chronic pressure overload or MI [204]. Interestingly, combined deficiency of PKC*δ* and PKC*ε* enhanced pressure overload-induced cardiac hypertrophy likely through disinhibition of ERK1/2 [205]. It is also known that overexpression of dominant-negative PKC*ε* but not other isoforms induced apoptosis in cultured primary cardiomyocytes, suggesting that PKC*ε* is required to maintain cell viability [203]. One potential mechanism underlying PKC*ε*-mediated protection is by inhibiting calcium-sensing receptor (CaR), a GPCR necessary for Ca^2+^ release from the ER [206]. PKC*ε* may also inhibit apoptosis by phosphorylating Connexin-43 at Ser262 and inhibit interaction between Connexin-43 and Kir6.1, a pore-forming subunit of ATP-sensitive potassium channels [207]. Another PKC isoform, PKC*θ*, has been shown to be protective based on the observation that global deletion of PKC*θ* led to p38/JNK activation and ventricular dilation after pressure overload, and PKC*θ* ^−/−^ cardiomyocytes were more susceptible to apoptosis upon stimulation with PE and hypoxia [208]. Treatment with PKC inhibitor chelerythrine or BIM-1 abolished fibroblast growth factor-2 (FGF-2) mediated protection against DOX-induced apoptosis, suggesting that PKC activation is an important prosurvival mechanism downstream of FGF-2 [209]. Interestingly, treatment with Ly333531, a specific PKC*β* inhibitor, attenuated mitochondrial depolarization and apoptosis in response to alcohol and advanced glycation end product, indicating a proapoptotic role of PKC*β* [210, 211].

## 10. PKA

Cyclic AMP-dependent protein kinase (PKA), the first protein kinase with its amino acid sequence and crystal structure being defined [212, 213], has served as the prototypical kinase for the entire eukaryotic protein kinase superfamily (kinome). The inactive PKA holoenzyme is a heterotetrameric complex composed of 2 regulatory and 2 catalytic subunits [214]. Upon hormone stimulation and GPCR activation, the *α* subunit of G protein binds adenylyl cyclase (AC) to convert ATP into cAMP, which then associates with the regulatory subunits and changes its conformation, eventually leading to release and activation of the catalytic subunit of PKA. In cardiac myocytes, PKA is best known for its role in mediating *β*-AR-induced contraction through phosphorylation of proteins involved in excitation-contraction coupling, such as L-type Ca^2+^ channels, ryanodine receptor, phospholamban, and cardiac troponin I [215]. Activation of PKA leads to feedback inhibition of its activity by enhancing phosphodiesterase 3B- (PDE3B-) dependent cAMP degradation into AMP and PI3K p110*γ*-dependent *β*-AR internalization [216]. This negative feedback mechanism likely explains earlier observation that chronic stimulation of *β*1-AR induced cardiac myocyte apoptosis through a PKA-independent mechanism that involves the Ca^2+^/calmodulin kinase II (CaMKII) [142]. It has been reported most recently that reduced *β*1-AR content on the membrane and decreased PKA activity in the cytoplasm and myofilaments account for impaired myocardial contractility in the diabetic mice [217].

In addition to enhancing contraction, PKA has also been implicated in other biological processes in cardiomyocytes, such as inhibition of hypertrophy [218] and potentiation of apoptosis [219]. Specifically, activation of PKA by catecholamine treatment or deletion of the PKA regulatory subunit I*α* (PKARI*α*) induced apoptosis by repressing the prosurvival function of myocyte enhancer factor 2 (MEF2) [218, 220] or by enhancing CREB-binding protein (CBP) and c-Myc-dependent transcription of Bim [219]. Moreover, hypoxia/reoxygenation-induced apoptosis is mediated by downregulation of PKARI*α* and consequent activation of p90 ribosomal S6 Kinase 1 (RSK1) [221]. In consistence with these findings, knockdown of the cAMP-hydrolyzing PDE3A was sufficient to induce apoptosis in cultured primary cardiac myocytes [222], possibly through PKA-dependent stabilization of inducible cAMP early repressor (ICER) [223]. Angiotensin II or isoproterenol stimulation induced PDE3A downregulation and myocyte apoptosis, which was completely blocked by overexpression of PDE3A, suggesting a critical role of PDE3A-dependent PKA inactivation in protection against apoptosis [222]. Similar to PDE3A, another isoform, PDE4D, appears to be cardioprotective as mice deficient in PDE4D developed cardiac dysfunction and accelerated heart failure following MI [224]. Intriguingly, cardioprotective roles of PKA have also been reported. For example, transient (30 min) treatment with nitrite induced PKA activation, leading to mitochondrial fusion and cytoprotection through phosphorylation of Drp1 at Ser655 [225]. Most recently, Lin28-induced inhibition of apoptosis in a diabetic cardiomyopathy model was shown to be abolished by pretreatment with a PKA inhibitor H89 [226]. These findings suggest that the role of PKA in apoptosis regulation is context dependent.

## 11. Cell Cycle Regulators

Mammalian cardiomyocytes exit the cell cycle and become terminally differentiated shortly after birth. However, expression of the cell cycle regulatory machinery including cyclin-dependent kinases (CDKs) and CDK inhibitors (CDKIs) persists into adulthood [227]. The catalytic activity of CDKs requires interaction with cyclins, which are regulatory proteins that also control substrate specificity. Emerging evidence indicates that both CDKs and CDKIs are multifaceted proteins with functions beyond cell cycle regulation [228]. For example, overexpression of dominant-negative CDK2 blocked apoptosis of cardiomyocytes in response to hypoxia stimulation, suggesting that activation of CDK2 is necessary for hypoxia-induced apoptosis [229, 230]. The underlying mechanism was believed to be the fact that CDK2-dependent hyperphosphorylation of retinoblastoma protein (Rb) disrupted Rb-E2F interaction, leading to E2F-mediated transcription of proapoptotic genes [230]. Interestingly, Rb hyperphosphorylation was not mediated by CDK4/6, and overexpression of dominant-negative CDK4/6 failed to protect against hypoxia-induced apoptosis [230]. These data are in agreement with recent findings that the early G1 phase cyclin D:CDK4/6 complex enhanced Rb-E2F interaction by monophosphorylating Rb, whereas late G1 phase cyclinE:CDK2 complex abolished Rb-E2F binding through Rb hyperphosphorylation, which were then maintained by the S phase cyclin A:CDK2 and M phase cyclin B:CDK1 complexes through S, G2, and M phases [231]. Indeed, protein level of cyclin A is gradually increased in response to hypoxia stimulation, and ectopic expression of cyclin A induced apoptosis in cultured cardiomyocytes [229]. Suppression of cyclin A-associated kinase activity by the CDKI p21 mediates the antiapoptotic effect of nitric oxide [232].

The E2F family transcription factors, usually sequestered and inhibited by Rb protein through Rb-E2F interaction, are classified into transcription activators (E2F1, E2F2, and E2F3A) and repressors (E2F3B and E2F4-8). E2F family members are best known for their role in G1-S transition but are also important regulators of apoptosis. Adenoviral delivery of E2F1 to primary rat cardiomyocytes evoked DNA synthesis and p53-independent apoptosis that is associated with activation of CDK1/2 and CDK4/6 and downregulation of CDKIs p21 and p27 [233, 234]. E2F1-induced activation of the intrinsic apoptosis pathway has been attributed to transcriptional upregulation of its target gene Bnip3 [235], which is necessary for I/R-induced apoptosis [236] and doxorubicin-induced necrosis [237]. Consistently, E2F1 null mice were protected against I/R-induced cardiomyocyte apoptosis with decreased expression of Bnip3 [238]. It is noteworthy that the expression of classical E2F1 target genes including caspase 3 and Apaf-1 was not reduced in E2F1 null mice either during development or after I/R injury [238, 239]. By contrast, the transcription repressor subfamily member E2F4 was able to inhibit transcription of proapoptotic genes E2F1 and Apaf-1 and antagonize hypoxia-induced cardiomyocyte death [240].

Based on their structure and CDK specificity, CDKIs are classified into the INK4 family CDKIs (p16^INK4a^ (Cdkn2a), p15^INK4b^ (Cdkn2b), p18^INK4c^ (Cdkn2c), and p19^INK4d^ (Cdkn2d)) that inhibit CDK4/6 activity by competing with cyclin D to bind CDK4/6 and the Cip/Kip family CDKIs (p21^Cip1^ (Cdkn1a), p27^Kip1^ (Cdkn1b), and p57^Kip2^ (Cdkn1c)) that associate with both cyclins and CDKs and interfere with the activities of cyclin D-, E-, A-, and B-CDK complexes. Consistent with the finding that activation of CDK4/6 is not required for hypoxia-induced apoptosis, overexpression of the CDK4/6-targeting CDKI p16 did not inhibit cardiomyocyte apoptosis in response to hypoxia stimulation [230]. In contrast, the Cip/Kip family CDKIs have been shown to play a critical role in regulating apoptosis. For example, protein levels of p21 are decreased in hypoxic- or doxorubicin-challenged cardiomyocytes [107, 229], and overexpression of p21 protected against hypoxia-induced apoptosis, an effect that may be independent of its CDK inhibitory function [230]. Using gain- and loss-of-function approaches, we recently showed that p21 represses expression of the proapoptotic BH3-only protein Bim and antagonizes doxorubicin-induced cardiomyocyte apoptosis [107]. p21 has also been implicated in mediating protection against oxidative stress downstream of the mitochondrial helicase, TWINKLE [241]. There is evidence that another Cip/Kip family member p27 is also protective in cardiac myocytes as p27 deficient mice exhibited increased apoptosis and infarct size following MI [242]. MI-induced cardiomyocyte apoptosis was attenuated by intravenous delivery of p27 fused with transactivator of transcription (TAT) to facilitate transport across plasma membrane [242]. The beneficial effect of p27 was mediated, at least in part, by activation of autophagy [243]. Interestingly, mice deficient in p21 or p27 have also been shown to be protected from some pathological conditions [244, 245]. This discrepancy may be explained by the fact that the mice used in these studies are germline knockout animals and thus many types of cells were affected instead of one single cell type. In agreement with the early observation that mice deficient in p57 displayed increased apoptosis in the heart and died shortly after birth [246], cardiac-specific transgenic expression of p57 attenuated ex vivo I/R injury [247], indicating a cardioprotective role of p57.

## 12. Jagged/Notch Signaling

Notch signaling regulates embryonic cell fate determination and adult tissue homeostasis through local cell-cell interactions. Mammalian cells express five Notch ligands (Delta-like1, Delta-like3, Delta-like4, Jagged1, and Jagged2) and four Notch receptors (Notch1–4). Upon binding with the ligand on the signal sending cells, the single-pass transmembrane Notch receptors on the signal receiving cells undergo proteolytic cleavage by *γ*-secretase, leading to translocation of the Notch intracellular domain (NICD) into the nuclei and transcription of downstream target genes (Figure 1). Myocardial Notch1 levels are decreased with postnatal development but are reactivated following injury [248]. Activation of Notch1 in the heart by either transgenic expression or intramyocardial injection augmented antiapoptotic signaling Akt and Bcl-2 and improved cardiac performance following MI [248, 249]. Ischemia pre/postconditioning-dependent protection against apoptosis is mediated by Notch1 activation [250, 251]. Cardiac-specific deletion of Notch1 increased the number of apoptotic cardiomyocytes following hemodynamic overload [252]. Interestingly, cardiac-specific Notch1 deletion did not affect post-MI cardiac repair possibly due to compensation by Notch2 and Notch3 [253]. Indeed, inactivation of Notch signaling by a *γ*-secretase inhibitor dramatically induced apoptosis, which was fully rescued by ectopic expression of the activated Notch1 NICD [254, 255]. Lentivirus-mediated overexpression of Notch3 has also been shown to protect cardiomyocytes from I/R-induced apoptosis [256]. It is noteworthy that Notch2-induced cell cycle progression resulted in DNA damage checkpoint activation and cell cycle arrest at G2/M in neonatal cardiomyocytes (postnatal day 5) [257]. Release of these cells from cell cycle arrest by treatment with caffeine (an inhibitor of ataxia-telangiectasia mutated (ATM) and ATM and Rad-3 related (ATR) kinases) dramatically induced an 87-fold increase in apoptosis [257]. Intramyocardial injection of the Notch1 ligand Jagged1 induced Notch1 activation and protected against I/R injury [258]. In contrast to full length Jagged1, the cleaved Jagged1 intracellular domain (J1ICD) inhibits Notch signaling through binding with NICD and promoting its degradation [259]. Cardiac-specific J1ICD transgenic mice exhibited decreased Notch activity, alleviated cardiac hypertrophy, and apoptosis in response to pressure overload [260]. Whether the inhibition of apoptosis is a direct effect of J1ICD or a secondary response to reduced hypertrophy is not clear at this point.

## 13. Calcineurin/CaMKII

Calcium signaling as a second messenger system regulates a number of biological processes in cardiac myocytes including excitation-contraction coupling and cell survival and death. Intracellular calcium ions (Ca^2+^) bind the calcium sensor calmodulin (CaM) in the cytosol and activate the Ca^2+^/CaM-dependent phosphatase calcineurin (also known as protein phosphatase 2B or PP2B) and the Ca^2+^/CaM-dependent kinase II (CaMKII), leading to changes in cell behavior. It is known that calcineurin binds Ca^2+^/CaM with high affinity (*K*_*d*_ = 2.8 × 10^−11^ M) and is activated by moderate levels of [Ca^2+^], whereas CaMKII is relatively insensitive to Ca^2+^ signals (*K*_*d*_ = 3.4 × 10^−8^ M) and is predominantly activated by a large elevation of [Ca^2+^] [261].

Cardiac calcineurin is best known for its prohypertrophic role through dephosphorylation of nuclear factor of activated T cells (NFAT), leading to its nuclear translocation and transcription of hypertrophic genes. Interestingly, persistent activation of calcineurin also protected against apoptosis (Figure 1) [262], and depletion of calcineurin A*β* augmented apoptosis through an NFAT-dependent mechanism in murine models of I/R injury [263] or dilated cardiomyopathy [264]. Moreover, calcineurin activation inhibited hydrogen peroxide-induced apoptosis [265], and selective inhibition of calcineurin-dependent NFAT activity induced apoptosis after treatment with phenylephrine [266], suggesting that calcineurin/NFAT is antiapoptotic in these settings, likely through upregulation of *α*-crystallin B [267]. However, cardiac-specific transgenic expression of a dominant-negative mutant of calcineurin attenuated cardiomyocyte apoptosis induced by isoproterenol infusion [141]. In this regard, calcineurin has been shown to induce apoptosis by dephosphorylation and subsequent activation of the proapoptotic protein BAD [268] and apoptosis signal-regulating kinase 1 (ASK1) [269] or by repressing AMP-activated protein kinase- (AMPK-) dependent autophagy [270]. Further investigations are needed to address the inconsistence between these studies.

Despite the fact that CaMKII*δ*B, the predominant nuclear isoform of CaMKII, has been shown to antagonize doxorubicin-induced cardiotoxicity [271], the majority of studies indicate a detrimental role of CaMKII in the heart. For example, activation of CaMKII is both sufficient and necessary for *β*1-AR-dependent cardiomyocyte apoptosis [142, 272]. Deletion of CaMKII*δ*, the predominant cardiac isoform of CaMKII, attenuated ventricular dilation and myocyte apoptosis following transverse aortic constriction and delayed transition to heart failure [273]. The mechanisms underlying CaMKII-mediated apoptosis have been attributed to direct phosphorylation of calcineurin at Ser197 [274] and subsequent repression of calcineurin/NFAT signaling [275], enhancing voltage-gated Ca^2+^ channel- (Ca_*V*_1.2-) dependent calcium overload [276], or phosphorylation of mitochondrial Ca^2+^ uniporter (MCU) at Ser57/Ser92 and increasing MCU current, leading to mPTP opening and cell death [277]. In addition, activation of CaMKII also contributes to myocardial I/R injury through promoting inflammatory signaling mediated by NF-*κ*B [278] and chemokine (C-C motif) ligand 3 (CCL3) [279]. Intriguingly, CaMKII inhibition with a small-molecule inhibitor KN-93 or the CaMKII inhibitory peptide not only protected myocytes against apoptosis but also inhibited necrosis following myocardial I/R injury [280]. Indeed, a most recent study revealed that phosphorylation and oxidation of CaMKII mediate regulated necrosis downstream of RIPK3 [281].

## 14. Progress in Clinical Translation

Two decades have passed since the first observation of myocyte apoptosis in human heart failure caused by ischemic heart disease or idiopathic dilated cardiomyopathy [282–285]. Cardiomyocyte apoptosis was later shown to be present in human immunodeficiency virus cardiomyopathy [286] and heart transplants [287] and following retrograde cardioplegia [288]. Growing evidence suggests that augmented myocyte apoptosis plays a causal role in heart failure pathogenesis, since induction of apoptosis by caspase 8 activation in mice to ~10–20% of the levels observed in failing human heart resulted in lethal dilated cardiomyopathy, which can be fully rescued by administration of a pan-caspase inhibitor [289]. However, to date, no drug that specifically blocks apoptotic pathways has been approved by the US Food and Drug Administration in the treatment of heart disease.

Erythropoietin (EPO), a hematopoietic hormone that can activate the PI3K/Akt, MAPK, PKC, and NF-*κ*B antiapoptotic pathways, has been shown to be cardioprotective in a variety of MI and I/R animal studies and small clinical trials [290–292]. However, larger randomized phase II trials REVEAL [293] and HEBE III [294, 295] failed to show any significant improvement in heart function and, instead, EPO treatment was associated with an increase in infarct size among older patients. Based on these findings, the effectiveness of EPO in the treatment of coronary artery disease has been questioned [296–298].

Experimental studies revealed that ischemic pre/postconditioning protects against cardiomyocyte apoptosis by activation of PI3K/Akt [24, 25, 46], Pim1 [55, 56], and Notch1 [248, 249] and inhibition of p38 [89, 90, 93]. Due to its clinical relevance and feasibility, postconditioning has been evaluated in more clinical trials and has been shown to reduce infarct size in patients with acute ST-segment elevation myocardial infarction (STEMI) [299, 300]. Interestingly, ischemic postconditioning in the heart also ameliorated acute kidney injury in patients with non-ST-segment elevation myocardial infarction [301]. It has also been reported that postconditioning did not seem to improve myocardial reperfusion [302, 303] or even showed a nonsignificant increase in infarct size in STEMI patients [304]. The discrepancy may be explained by differences in enrollment criteria, timing of treatment and evaluation, and endpoint selection. More rigorously designed clinical trials are warranted to further clarify the effect of postconditioning in STEMI patients.

## 15. Conclusions and Perspectives

Our knowledge regarding mechanisms underlying cardiomyocyte apoptosis has expanded enormously in the past decade with rapid advances in molecular techniques. We sincerely apologize to researchers whose work could not be covered. A plethora of genes and signaling pathways as reviewed above have been suggested to either promote or suppress cell death in the pathogenesis of heart disease. However, given the scale of human genome and the complexity of disease progression, it is very likely that a number of important apoptosis-regulating genes remain unidentified. High-throughput genome-wide screening coupled with in vitro/in vivo studies will greatly facilitate the discovery of novel drug target in apoptosis-associated heart disease.

Despite the fact that a large number of caspase inhibitors have been developed, none of them has been proved to be successful in clinical trials. A key explanation is that caspase inhibitors block apoptosis at late stages when severe damage such as mitochondrial outer membrane permeabilization has been executed and at this point the cells are committed to die. As a result, treatment with caspase inhibitors often leads to a morphologic shift from apoptotic to necrotic cell death and rarely confers long-term cytoprotection. Therefore, we believe that future intervention procedures should target upstream events at early stages of apoptosis to preserve mitochondrial function and to truly prevent cell death.
